# Beyond Value in Moral Phenomenology: The Role of Epistemic and Control Experiences

**DOI:** 10.3389/fpsyg.2019.02430

**Published:** 2019-10-30

**Authors:** James F. M. Cornwell, E. Tory Higgins

**Affiliations:** ^1^Department of Behavioral Sciences and Leadership, United States Military Academy, West Point, NY, United States; ^2^Department of Psychology, Columbia University, New York, NY, United States

**Keywords:** morality, judgment, epistemic feelings, truth motivation, control motivation

## Abstract

Many researchers in moral psychology approach the topic of moral judgment in terms of value—assessing outcomes of behaviors as either harmful or helpful, which makes the behaviors wrong or right, respectively. However, recent advances in motivation science suggest that other motives may be at work as well—namely truth (wanting to establish what is real) and control (wanting to manage what happens). In this review, we argue that the epistemic experiences of observers of (im)moral behaviors, and the perceived epistemic experiences of those observed, serve as a groundwork for understanding how truth and control motives are implicated in the moral judgment process. We also discuss relations between this framework and recent work from across the field of moral psychology, as well as implications for future research.

A good deal of recent research on the psychology of morality assumes that moral judgments turn on questions of *value*: a view that morality always, in some sense, constitutes a question of benefits or harm to the self, others, or community. This approach is particularly prevalent among social, cognitive, and evolutionary psychologists, and tends to rest on both a broad and particular focus of the moral judgment process. Evolutionary psychologists tend to turn to value to help explain morality’s existence in terms of the communal benefits it provides (e.g., Sober and Wilson, 1999). Among social and cognitive psychologists, the question is more focused on the content of moral judgments themselves. One expression of this approach is the notion that questions of morality center around a dyadic form of agent and patient (Gray et al., 2012), with rightness and wrongness, praise and blame following questions of agents helping or harming patients, respectively. Indeed, from a very young age, human children appear capable of distinguishing between “helpful” and “harmful” social entities, and show a clear preference for the former over the latter (Hamlin and Wynn, 2011). Regardless of the content of morality, whether it be questions of justice (Kohlberg, 1969), or broader questions about loyalty, authority, or purity (Graham et al., 2011), the key assumption appears to be that moral judgments are associated with declarations that certain actions are helpful or harmful, either intrinsically or in their effects. While this view is not universal among moral psychologists, if one might conclude from it that moral motivation is centered on determining the value of behaviors in terms of their resultant effects, and is straightforwardly aimed at maximizing benefits while minimizing harms (indeed, some psychologists who are ethical consequentialists make this claim explicitly, see, e.g., Pinker, 2002; Greene, 2014). This perspective emphasizes feelings stemming from judgments of value in the phenomenology of moral judgment.

We do not disagree that questions of value (i.e., wanting to have desired results) are very important in moral phenomenology. But we also believe that value *per se* only captures part of the picture of moral experience. In this review, we argue that many findings in moral psychology research also reflect the motives to establish what is real (truth motivation) and to manage what happens (control motivation). As for judgments and choices in general (see Higgins, 2012), truth and control motivations make contributions in the moral domain beyond just value motivation (beyond pleasure and pain). We argue, therefore, that future research should pay more attention to the truth and control motives of observers when examining the phenomenology of moral judgment. In particular, what needs fuller consideration is the role of truth or epistemic experience in moral phenomenology. By “epistemic feelings” we mean the intuitive sense that certain things are true or false, and the motivation to create a shared reality with others about those feelings (see Hardin and Higgins, 1996; Echterhoff et al., 2009; Higgins, 2019a). We believe that the epistemic experience of wanting to establish what is real is critical to the phenomenology of moral judgment.

Motivation science has distinguished between three fundamental motives. Value motives (wanting to have desired results) have been the major focus of research in motivation, and they form the basis of the notion that people generally want to approach pleasure and avoid pain (Franks and Higgins, 2012; Higgins, 2012; Cornwell et al., 2014). Some lines of research have even suggested that a straightforward tie can be made between motivation and morality by simply attaching approach motives to helpful, positive, moral behaviors and avoidance motives to harmful, negative, immoral behaviors (e.g., Janoff-Bulman and Carnes, 2013).

This perspective has been useful in exploring the contours of moral judgment. However, looking at valued outcomes exclusively can cause the incorporation of other elements into moral judgments to seem irrational or puzzling, such as incorporating intentionality (see, for example, the characterization of doing so in Ames and Fiske, 2013). Alternatively, the additional elements can be seen as serving motives other than valued outcomes. Indeed, as will be developed below, introducing other motives in moral judgment allows elements that feature prominently in other streams of moral judgment research, as well as older understandings of moral psychology to be reincorporated (e.g., Piaget, 1932/1997), including intentionality, culpability, recidivism, and justification, thereby broadening our conception of moral judgment.

Specifically, the value-only perspective neglects two important components of motivation, each of which operates independently of outcomes or results *per se*. The first, truth motivation, is about establishing what is real, correct, true, or right. Humans and other animals have a fundamental motive to understand and make sense of their environment, irrespective of specific outcomes of that understanding (e.g., curiosity). The second, control motivation, is about managing what happens. That is, humans and other animals have a fundamental motive to affect and shape their environments, whether that shaping is instrumental or not (Franks and Higgins, 2012; Higgins, 2012; Cornwell et al., 2014). Truth motivation and control motivation are intrinsically motivating. They are not simply instrumental extrinsic motives in the service of approaching good outcomes and avoiding bad outcomes. Indeed, for the sake of establishing what is real or managing to make something happen, people will endure pain and even risk death.

In this review, we argue that truth and control motivations of observers can come into play when judging the actions of others independently of the positive or negative results of the actions in question. In so doing, we integrate these motivational experiences into existing models of moral judgment. We will also highlight research findings from the last half century of moral psychology that we believe reflect the presence of truth and control experiences in moral judgment. Finally, we close with the case example of the American criminal justice system to illustrate how these motives play out in the procedural judgments of others, and we suggest potential areas for future research.

## Truth and Control Motives in Forming Moral Judgments

From a perspective that emphasizes valued outcomes, one could conceptualize the motivation to determine the rightness or wrongness of another’s behavior as wanting to assess the general contribution of that behavior to approaching benefits and avoiding harms. We argue—and believe that convergent evidence in moral psychology research suggests—that such questions of value are only part of the motivational experiences involved in how human beings actually go about making moral judgments. Moral judgments are also about: (1) the experience of establishing which norms, values, and beliefs are true or right; and (2) the experience of managing what happens in a social world. The former motivation involves *truth* or *epistemic* experiences; the latter involves *control* experiences.

How is it that observers’ truth and control experiences are implicated in moral judgments of others’ actions? This is perhaps best understood when thinking about immoral actions. With respect to truth motivation, immoral actions are not only likely to bring about negative consequences for someone, but they also implicitly challenge the established socially verified order. Just as norms tell us something about what sorts of behaviors are to be expected in a given context, immoral actions can communicate *information*. If intentional (i.e., it is clear that the actor *means something* by his or her action), these actions can declare that the actor does not believe that the rules are correct, or that he has found some exception to them. This, in turn, creates a need in observers to resolve this challenge to their understanding of reality, which creates an experience of epistemic need. This motive is not simply about avoiding negative consequences, e.g., a harmless action can nevertheless be deemed immoral, it is also about establishing what is correct or right.

The role of control motivation in moral judgments is also important. Again, looking at immoral actions, wrong behaviors can bring about unexpected effects that disrupt the flow of things and stir up chaos. This can create the phenomenological experience of a failure of control, a failure to manage not only another’s actions but another’s beliefs about what is right and wrong. Control feedback is needed to manage the other’s epistemic beliefs about what is right and wrong. This management comes about in the form of *blaming* the individual for the outcome of his or her behavior in order to try to steer his or her epistemic feelings back into line with the moral order that the observer considers epistemically satisfying.

Thus, we see that moral judgments address immoral behaviors of others by: (1) reaffirming the observer’s established epistemic beliefs of right and wrong by evaluating such actions as “wrong;” and (2) managing the others’ epistemic beliefs about right and wrong by giving feedback to the actors that such actions are “wrong” (e.g., expressions of blame)—an attempt to reestablish the predictable social order by attaching blame in order to attempt to bring about change in the actors’ moral epistemic beliefs. We can see this most evidently in punishment, which flows from declaring actions wrong and blameworthy, thereby establishing one’s own sense of what is right (*truth*) and managing others’ beliefs about what is right (*control*)—in each case satisfying a motivational need experienced phenomenologically.

Interestingly, working from a different direction, Cushman (2008) argues that moral judgments involve two separate mechanisms. The first mechanism begins with the action itself and then assesses the mental state of the individual performing the action in order to determine its rightness or wrongness, and the second mechanism begins with the consequences and tracks them back to the causal actor in order to assess his or her praiseworthiness or blameworthiness. Cushman and Young (2011) also demonstrated that many patterns found in the moral judgment literature (e.g., action/omission distinctions; means/side-effect distinctions) exist in judging non-moral actions as well. In this review, we argue that the motives underlying these two mechanisms are truth motivation and control motivation, respectively, and not that these convergences highlight some potential ways in which truth and control motivations can be relevant to important lines of research in moral psychology. In the next two sections, we unpack these two processes, and highlight research suggesting that these motives are at work in the formation of moral judgments.

## Truth Motivation: Establishing What is Right

The first component maps onto truth motivation. By engaging in behaviors deemed “wrong,” an individual symbolically actualizes an alternative worldview, which will create an experience of an epistemic need in observers of the action. That observer might ask, “What did he *mean* by that?” If, for example, I steal from a neighbor, I am not merely harming the neighbor by removing some property from him, but I am also implicitly challenging the correctness of our current understanding of property rights. In other words, the action *means* that I, as a thief, believe that the claim that my neighbor has over his property is *false* (and, by implication, that I am not acting immorally at all, even if I acknowledge that my actions are harmful to the other)—think Robin Hood. This challenge requires an epistemic response in observers to restore a sense of what is right and correct. The action can be *rationalized* in order to justify it according to an alternative set of moral principles. It can be emptied of its symbolic content by declaring the actor’s incapacity (e.g., “He’s just an infant and couldn’t possibly know any better”) or non-intentionality (e.g., “He didn’t mean to do that”) to make such a declaration. Otherwise, it must be authoritatively *judged to be wrong*. Regardless of the avenue taken, the experience of the motivation for establishing what is real, right, and correct drives individuals to find a way to *make sense* of the action and provide a judgment.

Note, however, that in order to engage in this aspect of motivated reasoning, the observer must consider whether the actor is making some kind of declaration by his or her behavior. Is the actor making some sort of truth claim? Intentionality of the actor has long been recognized as an important component of moral judgment formation (e.g., Piaget, 1932/1997; Berg-Cross, 1975; Sternlieb and Youniss, 1975). For the purpose of truth motivation, perceptions of intentionality are independent of the results of the action (Malle and Knobe, 1997). Adding the element of intention—while holding the harmfulness of the consequences constant—should make individuals see that action as *more* wrong, since it would then also concern the observer’s epistemic feelings. This is, in fact, what researchers have found. Among both adults (Ames and Fiske, 2013) and children (Karniol, 1978), intentional actions are deemed to be more wrong than unintended actions, even if the outcomes are identical.

This finding has been reinforced by neuroimaging studies as well. Young and Saxe (2009) found that the integration of intentions into moral judgments is associated with greater activation in the temporo-parietal junction. This region has been associated with the ability to understand the beliefs of others in both moral (Young et al., 2007) and non-moral (Perner et al., 2006) situations. If moral judgments were entirely about the implications of the *outcomes* of the behavior for observers, then whether or not the action was intentional or not should not influence judgments of how wrong it is. Indeed, other neuroscience research examining those with damage to another part of the brain—the ventromedial prefrontal cortex—has shown that those with damage to this region fail to integrate intentionality information into their moral judgments; that is, they reason about the wrongness of behaviors *entirely* from the consequences of those behaviors (Ciaramelli et al., 2012), and they behave in a more utilitarian manner when forced to choose in dilemmas asking people to perform an immoral action to bring about good consequences (such as pushing a man off a bridge to save five others from an oncoming trolley, Koenigs et al., 2007). In fact, those with damage to this region are more likely to judge intended, but unsuccessful, harms (such as attempted murder) as more morally permissible compared to healthy controls (Young et al., 2010).

Given that intending to bring about a negative outcome provides information about an individual’s character, and, by extension, information that helps an observer manage what happens in his or her social environment, might the question of intention be a matter of *control* rather than truth? The possibility that these effects are due entirely to control motives and not to truth motives belies the fact that people are willing to mitigate their judgments of the wrongness of others even when those individuals still pose a danger—and perhaps even a *greater* danger—compared to someone who intends the behavior. For example, research has shown that people will respond less aggressively to an attack from someone who is maladjusted than they do from a normal person (Jones et al., 1959), presumably because they did not interpret the action as intentional in the former case. In this case, the harms were identical, and, if anything, the threat to one’s ability to manage what happens in the social environment is *greater* in the “maladjusted” individual’s case, but nevertheless the action was treated as less intended, and therefore less wrong, and therefore less meriting of a response in kind. From our perspective, the maladjusted person is treated as incapable of authoritatively declaring that his behavior is correct or right in this case, and thus is not making any declarations about the truth or falsehood of shared beliefs of the community with regard to acceptable behavior. Given this, an epistemic need in the observer is not created.

In addition to the above example, research has shown that sometimes individuals do not merely *mitigate* harmful actions, but also *justify* them. That is, they incorporate ambiguous actions into their moral understanding of what is truly right. For example, sociological research has shown that a common way for an individual to justify stealing is to rationalize an explanation for how the things stolen are actually rightfully his, and were stolen originally by their current owner (Sykes and Matza, 1957). If morality is merely about getting good results and managing what happens, then it is odd that there would be a motivation to justify obviously harmful and disruptive actions, unless incorporating those actions into the existing communal values and norms would satisfy an epistemic motive of those who observe that action. When it comes to violence through war, governments frequently find moral justifications (such as communal self-defense) for engaging in such violence (Goldmann, 1971). Indeed, individuals generally perceive aggression as wrong, but change their views if that aggression is in self-defense (Carpenter and Darley, 1978)—the behavior and its consequences are identical, but what the behavior *means* is different.

This process is perhaps akin to the reduction of cognitive dissonance we see in the classic studies by Festinger and Carlsmith (1959), in which participants only experience uncomfortable dissonance if they cannot justify their false statements to subsequent participants. Telling others that a boring study was fun *means* something different if you are being compensated $1 (low justification) versus $20 (high justification). More recent research has shown that those in an Asch-like social influence experiment who make non-normative judgments of behaviors in public (e.g., calling murder “morally acceptable”) do not simply abandon those judgments in private. Instead, they integrate them into existing moral frameworks—they *rationalize* them. This suggests that there is a component to moral judgments that relies on epistemic experience (Cornwell et al., 2019).

Seeing truth motives as integral to the formation of moral judgments also helps to explain two other areas of research that do not obviously take on the agent-patient dyadic form of much of moral psychology research (Schein and Gray, 2018): harmless immoral behavior and symbolic moral protest. Research has shown that people are less likely to incorporate intentions into judgments of harmless impure actions (such as drinking urine) compared to harmful actions (such as murder, Young and Saxe, 2011), but they still have difficulty affirming the moral acceptability of these harmless impure actions. Research suggests that this derives from their perceived *weirdness* (Gray and Keeney, 2015). It could be that some harmless actions engage an observer’s epistemic motivations to resolve them through moral judgments simply because the actions themselves are so difficult to fit into their worldview of what makes sense generally. Indeed, results from earlier research suggests that a lack of familiarity with the stimuli leads children to be less likely to differentiate “moral” from “conventional” violations (Davidson et al., 1983), suggesting that there is an important, perhaps inadequately understood, epistemic component to moral judgment formation when encountering behaviors that do not make sense because they are totally outside of normal experience.

One piece of evidence suggesting such a process involves motivational moderation of judgments of the now infamous example of sibling incest in which a brother and sister, who, after taking several precautions to keep their act a secret and ensure against conceiving a child, decide to sleep together. In response to this scenario, observers could consult their epistemic intuitions about what sorts of behaviors good and decent people engage in and whether this incest behavior fits within that worldview, or observers could simply focus on the components of the incest action that are directly related to potential harm and reason that way. Research on the effect of regulatory focus on moral judgments has shown that individuals in a promotion state (a state in which individuals are more likely to incorporate feelings and intuitions into their judgments, see Avnet and Higgins, 2006), compared to those in a prevention state (a state in which individuals are more likely to need explicit reasons to render a judgment), judge acts of harmless incest more severely (Cornwell and Higgins, 2016). This may be because relying on intuitions might lead observers to consider the behavior in its entirety and how it fits into phenomenological epistemic experience, whereas relying on reasons may lead observers to focus only on the elements that they deem relevant for making moral judgments.

Further evidence that effects concerning harmless wrongs are due, at least in part, to an observer’s truth motivations comes from a finding from another analysis of Young and Saxe (2011). An individual is thought to be doing something less morally wrong if he intends to commit incest but it turns out he is not related to his partner, compared to a situation where he intends to commit incest, but he is simply unable to carry out the act due to some intervening circumstance (in this case, a fire alarm). Consider these conditions in terms of whether the individual was making a problematic truth claim that needed to be overturned. If planning incest itself is what challenges one’s conception of what is true or correct, then that would be better resolved by it turning out that the act would *not* have been incest rather than having what would have been incest simply not occur. The observer in the latter case has to deal with a planned act of actual incest that is making a problematic truth claim. This is consistent with research showing that even *thinking* about forbidden actions that transgress sacred taboos is morally aversive (Tetlock et al., 2000).

Moral protest movements are another aspect of moral judgment that can be helpfully illuminated by considering truth motives. Protests over political issues typically arise from a grievance—a belief that one’s self or one’s group is being *wronged* in some way (Van Stekelenburg and Klandermans, 2013), and organizers attempt to increase a sense of moral outrage among participants in order to increase participation (Van Troost et al., 2013). But *why* should an individual participate in a protest? It is possible that, for example, an individual may accomplish some goal through protesting—that is, the collective action of the group can possibly provide some desired outcome, thereby satisfying value motives. However, it is unlikely that any given protest will actually bring about some desired goal. One could, instead, look at political protest movements as a way for subgroups to try to establish an alternative reality to the dominant one within a culture. That is, people engage in moral-political protest not simply because they want to get something done, but because they want to express their epistemic feelings and declare what they believe to be *true*. Indeed, research has actually shown that *ideology*—belief that the policy being protested is *wrong* (truth)—as opposed to *instrumentality*—getting something out of the protest (value)—is the stronger motivator for protest participation (Van Stekelenburg et al., 2011). Thus, understanding the moral motives of political movements might benefit from the examination of epistemic feelings resulting from truth motivation.

This latter example is also useful for drawing a distinction between what we are and are not claiming with respect to moral judgment. Nothing we have argued about the importance of epistemic motives with respect to moral judgment in any way takes away the *content* of the moral judgmental processing, but instead shifts the focus on the *motivation* for that judgmental processing while referencing that content. For example, the issue of fairness is widely understood to be important to moral judgment (Kohlberg, 1969), and it is implicated in the example of moral protest we provided above, given that three of the four *ideology* items in those studies were measured by assessing the degree to which people believed the protested policy to be “unfair” or “unjust” (Van Stekelenburg et al., 2011)^1^. What concerns epistemic motives, however, is that it is the *belief* that these policies are unfair that is driving the moral protest: the protest is supposed to establish *as true* that the policies are unfair. The protest is not about attaining better outcomes (instrumental value); it is about establishing the truth. Notably, epistemic motives are typically surrounding other issues like questions of harm, fairness, loyalty, and purity. However, they do so as establishing the truth: the motivation to establish that an action is *really* harmful, is *truly* unfair, is *actually* disloyal, or *genuinely* violates standards of decency.

Although we have only discussed responses to immoral action because that has been the general focus of moral psychology research, truth motives are also at work in moral action as well. Actions motivated by moral considerations can also influence the epistemic feelings of observers because, by behaving in a moral way, people are declaring that certain things are *true*. This is particularly evident when the morally good behavior is non-normative (i.e., the implicit beliefs underlying the behavior are not necessarily shared by the general community). A vegetarian, for example, may declare that she believes it is morally wrong to eat meat, implying that non-human animals have rights akin to humans, and their bodies can therefore not be used for food. Non-vegetarians will need to find a way to address that challenge to their epistemic feelings. They may reject that eating meat is wrong, but to do so, they will need to *rationalize* their own behavior according to their principles (e.g., “Animals eat other animals, and humans are animals too, so why can’t I eat animals?”). Alternatively, they can *explain away* the other’s judgment by removing that person’s authority to make it (e.g., “Maybe that’s true for her, but not for everyone.”). Ironically, this non-normativity can be so threatening to an individual’s epistemic phenomenology, that it can *itself* be declared immoral (e.g., “She’s just a fussy busybody telling everyone how to live their life! She should be ashamed of herself!”). Research on non-normative exemplary behavior has shown that such behaviors do, in fact, produce derogation of actors by observers that do not share their values (Minson and Monin, 2012). If morality were simply about results or communal well-being, how could another person’s desire to hold himself or herself to “higher” moral standards be threatening?

In this section, we highlighted components of the moral judgment process that are closely tied to the motivation to establish what is real and right and true (truth motivation). Providing a greater space for the experience of truth motivations in understanding moral judgments can give motivational force to existing action-processing models of moral judgment, as well as provide a framework with which to understand justifications of behaviors otherwise deemed wrong, judgments against odd, impure behaviors that nevertheless harm no one, and motivations for participation in (sometimes hopeless) political protests. In the next section, we will look at another motivational experience—control (wanting to manage what happens)—and explain how it relates to moral judgments. In particular, we will focus on how it relates to moral blame or praise of others’ behaviors independently of the valued outcomes of those behaviors.

## Control Motivation: Managing What Happens

By creating disruption, disorder, or social chaos, immoral actions have the capacity to cause individuals or communities to lose the ability to manage what happens, and thus the source that is *responsible* for the unexpected situation needs to be identified and corrected in order to restore *control*. That is, one is no longer simply motivated to declare immoral actions as *wrong* because they have implicitly declared statements of fact about the world, but the persons who enact them need to be identified as *blameworthy* for those actions, so that a change can be brought about in *their* worldview and *their* epistemic feelings to bring them back in line with the observer’s worldview. They need to be given control feedback to manage their epistemic feelings.

Saying people are blameworthy (as opposed to just “wrong”) is to say that they require some kind of correction in their beliefs that underlie their behavior. Importantly, for an action to be blameworthy, the person who enacts it (who would be the recipient of the blame) needs to actually be the cause of the disruption, and needs to be capable of correction, otherwise the response would not actually provide a means of effective management—i.e., it would not serve control motives. Therefore, it is necessary to infer, either implicitly or explicitly, that the person deserving of blame actually believes in what he or she is doing and has a *capacity* to be able to follow the rules; that is, to have an epistemic experience of judging between multiple courses of action and a capacity to choose one option over other alternative options. All of these factors go into determining the actor’s *blameworthiness* or *praiseworthiness* for what he or she has done. Regarding immoral behaviors, blame provides a way that groups can make it clear—provide feedback—that the epistemic feelings underlying certain behaviors are wrong, thereby changing those epistemic feelings to be more in line with those of the community (Malle et al., 2014).

It is worth pointing out that control motives do not necessarily come into play in moral judgments in the way that truth motives do. It is also worth highlighting that, unlike judgments of moral wrongness, judgments of moral blameworthiness are more person-centered than action-centered (Malle et al., 2014). That is, claiming that the actor is responsible for an action is necessary when judging moral blameworthiness, but is not necessary for judging moral wrongness. An action can be unintentional and still morally blameworthy, but its blameworthiness is rooted in the desires (or lack thereof, in cases of negligence) and capacity of the actor to choose different courses of action.

This is well illustrated by examining an effect highlighted by the literature called the “side-effect effect.” In the classic version of the scenario that is used to show this effect, the Chairman of a company needs to choose between two courses of action. In the “harm” version of the scenario, choosing action A will lead to profits, but will harm the environment, whereas action B will do neither. In the “help” version of the scenario, choosing action A will lead to profits, and will also help the environment, whereas action B will again do neither. In both versions of the scenario, the Chairman makes it clear that he cares only about profits and does not care about the environment, and always chooses option A. In the former, “harm,” version of the scenario, choosing action A is considered morally blameworthy, whereas in the “help” version of the scenario, choosing action A is *not* considered morally praiseworthy (Leslie et al., 2006).

There have been a number of attempts at explaining this disparity. One school of thought looks at the two situations and notes that only in the “harm” scenario is there an obviously intentional trade-off that implies a causal link between the outcome of harm and the individual making the decision (Machery, 2008). That is, only in the “harm” version of the scenario is the Chairman’s character really implicated in the impact on the environment, and, therefore, only in the harm version is his epistemic feeling in need of correction. Other research (Guglielmo and Malle, 2010) suggests that it is, indeed, the perceived *epistemic feelings* of the actor (i.e., what he *desires*, or what he believes is *good* to do, to bring about) that leads to the judgments of blameworthiness for the action: there were multiple courses of action available and he chose the *wrong* one because he felt profits were more important than the environment. Other research has shown that perceived “metadesires” is what leads to actions being perceived as morally praiseworthy—moral actors can be implicitly understood as believing it is *truly good* to help others, and therefore, disavowals of the rightness of that action lead to reductions in this baseline level of praise (Pizarro et al., 2003). That is, there has to be a direct logical link between the observed outcome and the feelings of the actor in order to make a judgment that an action is morally blameworthy or praiseworthy, because that suggests a need for correction—an experienced need to bring about a change in the actor’s epistemic feelings. Again, this is consistent with the notion that motivations to manage what happens can influence our moral judgments; actions are only morally blameworthy or praiseworthy if their outcomes can find their source in an individual whose epistemic beliefs or feelings can be managed.

Why is the assessment of such epistemic beliefs or feelings important? In order to manage the social environment (either individually or collectively), it is necessary to know which individuals are good and which are bad. By using the meaning of different action options in ways that provide information about what people believe and feel, we can learn about their moral character and predict how they will behave in the future. This provides observers with a greater capacity to manage what happens, including using blame and praise to change that character (i.e., their beliefs and feelings). It is important to emphasize, however, that this management function is not purely instrumental in nature—the desire to manage the epistemic beliefs of individuals is not purely a matter of increasing the likelihood that they will benefit the community in some way and minimizing the likelihood that they will do harm. If this were the case, then blame and punishment would purely be motivated by deterrence, rather than by retribution, and research suggests that the opposite is true (Carlsmith et al., 2002). Furthermore, if such judgments were merely about maximizing and minimizing certain behavioral outcomes, then such judgments would not be influenced by things such as contrition or incapacity, both of which, we highlight below, are incorporated into the assessment of blame.

This social management function is a prominent feature of moral judgment, leading some researchers to adopt a more person-centered approach to morality (Uhlmann et al., 2015) because it helps to explain some otherwise puzzling evidence from the literature. For example, research has shown that people judge someone who caused a large automobile accident more harshly if they learn that he was speeding so he could hide his cocaine than they do if the speeder is rushing home to hide a present (Alicke, 1992). In a manner similar to the “side-effect effect” noted above, the outcome leads logically back to the person who caused it, and then the desires and feelings of that person are judged in order to determine his or her character, and then blameworthiness or praiseworthiness of the action is assigned. Note that the consequences act merely as a signal that gets the full process in motion; it is only after assessing that the character of the person necessitates moral management is blame or praise applied.

How are the feelings of the person inferred? Research has shown that one way people do this is to look at the emotions or quick decisions of a person. The emotions of an actor are considered to provide more useful information to establish the actor’s responsibility than the thoughts of an actor (Fedotova et al., 2011). Quicker decisions by an actor are perceived by observers as providing more insight into the character of the actor, leading to stronger negative judgments for immoral behavior and more positive character evaluations for moral behavior (Critcher et al., 2013), which can provide information concerning how he or she can be expected to behave in the future and what sorts of blame or praise is necessary to manage his or her motives. Other research has shown that even young children can do this. From infancy, children prefer helpers that appear to want to help than those who do so accidentally, and harmers that harm accidentally to those who appear to want to harm. Such preferences would be helpful in managing one’s social circle as one ages (Woo et al., 2017).

This also helps explain findings relating to blameworthiness attributed to actions that are, at least in part, caused by third parties. People generally will reduce the level of blame of an actor if a “higher order” causal actor can be the focus of the blame attribution (Phillips and Shaw, 2015). Those who are “just following orders” are given less blame than those who carry out actions on their own volition. However, it is worth noting that this effect can be attenuated if the proximal actor “identifies” with the action in question (Woofolk et al., 2006). That is, if an individual makes it clear that he or she believes that it is the right action, that person receives more blame. However, if that person is performing the action at the order of another, one cannot infer that the person believes it is the right action, and therefore one’s motive to manage epistemic beliefs or feelings to be in line with the right beliefs or feelings is better expended on the person truly responsible for the action.

These motives can provide an alternative understanding of cases where judgments are impacted by the nature of the outcome, rather than relying on an explanation that has to do with valued outcomes *per se*. For example, research has shown that individuals will provide more blame for bad outcomes that result from negligence in order to defend against the idea that they might be severely harmed by chance (Walster, 1966). That is, individuals are motivated to see their world as within their control, and as having outcomes that can be managed. Other research shows that people will increase the level of punishment for offenses if the crime in question is widespread and rarely punished (i.e., it is *out of control*) as a means of believing that such crimes can be brought back under social control—by potentially changing the epistemic feelings of those who observe the punishment (Tetlock et al., 2007). In these cases, it is not the outcomes or their magnitude *per se* that are the source of the blameworthiness judgments. Rather, it is the implications for believing that society can control individuals’ beliefs and, thereby, their actions. For example, research suggests that when conceptualizing moral rules as being “useful,” people are more likely to endorse them if they are motivated by a regulatory mode (i.e., locomotion concerns with effecting change) that is associated with control concerns (Cornwell and Higgins, 2014).

Other research suggests that the experience of control can act independently of other motivations, thereby influencing moral judgments in unexpected ways. There is evidence, for example, that observers’ own experience with the degree to which their own actions and behaviors reflect their will can impact the intensity of their judgments of others. Research shows, for instance, that those with a low subjective socioeconomic status are more likely to provide situational attributions in judging the actions of others, presumably because they themselves experience their own actions this way due to their repeated experience (Kraus et al., 2009). Other research suggests that, when using their intuitions in making judgments, individuals will intensify (*vs*. de-intensify) their judgments of others if they are currently experiencing a strong (*vs*. weak) sense of control (Cornwell and Higgins, 2019). This is consistent with other research suggesting that a component of the moral judgment process involves observers putting themselves in the position of the perpetrator and making judgments based on what they experience when doing so (Miller and Cushman, 2013).

What we are emphasizing here is that, although outcomes contribute to the assessment of blame or praise—indeed they appear to set the process in motion in many cases—blame or praise is *not* completely driven by outcomes. Instead, the blameworthiness or praiseworthiness of the *person* is crucial because it relates to whether that person’s epistemic experience about what is right and wrong can be managed. There is research, for example, showing that individuals treat actions as blameworthy even if they have no direct link to harms (Inbar et al., 2012). In this research, individuals who make an investment that is contingent on some major disaster hitting a third world country are judged as morally blameworthy. This is not because logically those individuals actually cause the major disaster, but because they reveal themselves to be people with moral beliefs and feelings that must be managed by applying blame. The logical connection between blame and outcomes, again, is quite secondary to questions of control motivation.

In this and the previous section, we have reviewed evidence from the literature highlighting aspects of the moral judgment process that are not only about responding to perceived benefits and harms for society from an action (i.e., positive and negative outcomes), suggesting that moral judgment is also about responding to the truth-related implications of the action for one’s own epistemic experience of what is right and wrong and the control-related implications of managing another person’s epistemic experience of right and wrong. Although we have reviewed a good deal of laboratory evidence here that illuminates how these processes play out in the phenomenology of people, it may be helpful to highlight how they play out socially in a public process: the American criminal justice system.

## The Case of the American Criminal Justice System

Although the truth and control motives are likely to be closely intertwined in everyday moral judgments, we see them somewhat disentangled in the American criminal justice system. During the initial phase of a trial, the jury is asked to render a *verdict* (a word coming from the Latin *verum dictum*—“true word”) on whether the behavior in question is actually illegal according to the standards laid out by the law. To achieve a “guilty” verdict, the prosecutors must establish that the defendant: (1) committed the act in question; (2) knew that the action was wrong at the time of commission; and (3) did not do it for a reason that would provide adequate justification. These latter two defenses are worth noting because they underscore the degree to which epistemic feelings come to bear upon a case. Assuming that the defendant did commit the crime in question, the only way that the action can be considered anything other than illegal is by *rationalizing* the action of the defendant according to an alternative set of moral principles (e.g., killing in self-defense or killing in defense of another) or by declaring the actor incapable of appreciating the wrongness of the actions (e.g., not guilty by reason of mental defect). That is, in order to merit criminal prosecution, establishing the harmfulness of an action is insufficient to confer guilt—the person must also have a *mens rea* (a “guilty mind”)^2^. Once these standards have been met, beyond a reasonable doubt, the jury then authoritatively establishes that the crime is real—the norm (i.e., epistemic feeling or belief about what is right) was really violated, and the defendant is *really* guilty of that violation.

Then comes the sentencing phase. Even if an action is wrong, how blameworthy is it? At this phase, the trial moves from the question of whether the defendant’s action was wrong to what the effects of this wrongful action were *and* to what extent would applying blame influence his epistemic experience, and that of others, about what is right and wrong. Judges in this phase can draw on victim impact statements, as well as other aggravating and mitigating factors such as whether this is the defendant’s first offense and whether he or she expresses remorse or regret. These factors signal whether that individual has the capacity and desire to follow the rules in the future; i.e., whether blame will actually manage what happens to the defendant’s epistemic experience. There is also the urgency of deterring the epistemic feelings and beliefs that can bring about these kinds of actions in the future by others; i.e., whether blame will actually manage what happens in society, particularly with respect to what others will think is acceptable by punishments given or not given.

Thus, what determines blameworthiness is rooted in the need for society to manage what happens to epistemic feelings and beliefs about right and wrong. The more it seems that severe punishment is necessary to change the epistemic feelings and beliefs about right and wrong in the actor, and in others, who might perform the same action in the future, the heavier the punishment will be. In contrast, many minor offenses, sincerely regretted and unlikely to be repeated, may get the minimum sentence possible because only a minor change is needed in the epistemic feelings and beliefs about right and wrong.

Although truth and control motives appear to be pulled apart in the two-part process of criminal prosecution, it should not be overlooked that they are mutually reinforcing, particularly from the perspective of an observer of the process. When people witness a crime, they become motivated to see that the criminal is brought back under control and done so authoritatively in line with their epistemic feelings. Indeed, people appear to be prepared to perceive moral words more than non-moral words (Gantman and Van Bavel, 2014), and this effect is diminished when exposed to just outcomes, suggesting a kind of satiation of motivation (Gantman and Van Bavel, 2016). Criminal trials can provide satisfaction of both truth and control motives by providing both (1) an authoritative declaration, from observers’ perspectives, on whether a behavior is right or wrong; and (2) an appropriate punishment for that behavior that attempts to manage, to change, others’ understanding of what is right or wrong.

In support of the first point, research has shown that individuals regard criminal justice outcomes as more fair when that outcome is brought about by a trial than by a vigilante when the guilt of the defendant is ambiguous (Skitka and Houston, 2001). That is, when it is uncertain whether a person *truly* committed a criminal act, a group of one’s peers provides a more trusted outcome than individuals taking matters into their own hands. In support of the second point, punishment seems to be largely explained by the perceived deservingness of individuals for the punishment received (Carlsmith et al., 2002), which is, again, rooted in an evaluation of the character of that individual as it relates to what is relevant for socially managing him or her. Finally, while judgments of wrongness appear to be determined by one set of factors in moral judgments, and judgments of blameworthiness another set, punishment appears to be determined by the combination of these factors (Cushman, 2008), suggesting that *both* truth and control motivations work together in the moral judgment process.

## Future Directions and Conclusions

We have argued in this review that, over and above considerations of outcomes (i.e., value), motivational experiences involved in establishing what is real and managing what happens underlie the processes of moral judgment of others. In particular, we have argued that observers’ own truth and control motivations influence their judgments of others rooted in the observers’ own epistemic feelings (truth) and their concerns with managing the epistemic feelings of others (control). Such a conceptualization provides a motivational framework that aligns with research on decoupling judgments of actions and consequences (e.g., Cushman, 2008) and research examining the importance of person-centered judgments in moral psychology (e.g., Pizarro and Tannenbaum, 2012). This framework provides potential connections between these processes and other motivational factors that may be at play in moral judgment. They also provide potential starting points for interesting research in related domains.

One area is the relation between morality and meaning in life. Research has shown that those whose lives are judged to be more meaningful are also more likely to be seen as having lives that are morally good (King and Napa, 1998). Indeed, people are hesitant to describe others as “happy” if their positive psychological states are the result of activities deemed less moral (Phillips et al., 2011) or outright immoral (Phillips et al., 2017). Other research suggests that perceiving moral goodness in others leads observers to believe that they have a better understanding of their “true self” (Christy et al., 2017). Indeed, other research has tied this notion of the “true self” to moral judgment (Newman et al., 2015). Future research could examine more closely the motives underlying these effects. For example, meaning in life has to do with coherence, significance, and purpose (Martela and Steger, 2016). When assessing these components, it is likely that individuals are also assessing what the implied truths of a life are, and the perceived praiseworthiness of that person’s desires. Perhaps probing components of meaning in life in light of truth and control motives could provide deeper insights into its relation to judgments of morality.

Another area of future study involves assessments of victims. “Blaming the victim” is commonly understood as a means by which individuals can both maintain that a world is really just (van den Bos and Maas, 2009) and maintain a sense that one is capable of managing what happens in that world (e.g., an internal locus of control, see Maes, 1994). Other recent, perhaps more puzzling, research has shown that individuals actually give *more* agency to inert victims of harm (e.g., patients in a persistent vegetative state, robots, corpses, see Ward et al., 2013). Understanding the motives that underlie these effects, which appear to relate to both truth and control motives in some way, may give us a better sense of how victim derogation occurs, and find solutions to it.

A final area of additional research might be a deeper exploration of how these processes arise during the course of human development. As noted above, many of these processing dynamics are already present early in childhood, but there is other work suggesting that children improve in their ability to distinguish between negligent versus non-negligent behavior and justifiable versus non-justifiable harm better when their parental discipline focuses on these kinds of distinctions (Darley and Zanna, 1982). What this suggests is that discipline not only has the capacity to help parents manage their children’s behavior, but that discipline (especially when combined with induction explanations) also teaches their children about the world, bringing the epistemic phenomenology of children into harmony with caregivers’ worldview (Higgins, 2019a).

Discipline serves epistemic purposes by teaching children what kind of world they live in; for example, a world of gains and non-gains or a world of non-losses and losses (Higgins, 2019a). This is true independent of the outcomes of that discipline. Another classic example of the importance of truth feedback is parental use of “inductive discipline” where contingency rules are given explicitly and reasons are given for the discipline (e.g., Hoffman, 1970; Higgins, 1991). For example, discipline aimed at orienting children and adolescents toward the plight of victims (as opposed to using techniques such as love withdrawal or power assertion) has demonstrated increases in empathy and prosocial behavior among children (Krevans and Gibbs, 1996) and stronger moral identity among adolescents (Patrick and Gibbs, 2012). This could also fold into a broader exploration of moral behavior, which has been found to be connected, in part, to epistemic motivations (Cornwell et al., 2017). It also offers an opening into exploring the degree to which descriptive truths and prescriptive truths, although both stemming from truth, function in different ways, both epistemically and socially (Zimmerman, 2015). Another area for both child and adult development involves situations in which an individual believes an action to be wrong, but nevertheless still commits the action. This may be an interesting area where both truth and control motives need to align. Future research needs to examine how the epistemic and control motives function interactively, distinctly, and in tandem.

While it is evident that moral judgment is motivated, in part, by the desire to maximize beneficial behaviors and minimize harmful ones, understanding moral judgements comprehensively requires their examination in light of other fundamental motivations—in this case, truth and control motivations. Human beings are not merely motivated to maximize benefits and minimize costs of others’ behavior. They are also motivated to personally affirm what is correct or right about the world, and to use blame and praise effectively to manage what others believe is correct or right about the world—the epistemic feelings of self and others. Both the truth motivation and the control motivation underlying moral judgments are in the service of regulating epistemic feelings and beliefs—one’s own and those of others. In this sense, it is all about wanting to establish and maintain epistemic truth. In the moral domain, and in other life domains as well, value matters and control matters, but, ultimately, truth, and especially shared truth, reigns supreme (Higgins, 2019a,b). Examining moral judgment in light of these epistemic experiences provides additional complementary support for existing models of moral judgment, and provides several avenues for future research by moral psychologists.

## Author Contributions

The authors jointly devised the theoretical thrust of the review article. JC wrote the first draft. JC and EH continued to add to and revise it to produce the final draft submitted here.

### Conflict of Interest

The authors declare that the research was conducted in the absence of any commercial or financial relationships that could be construed as a potential conflict of interest.

## Footnotes

^1^We note that one of the items, “I want to take my responsibility,” more closely aligns with control motivation, so the connection is not perfect.

^2^There exist “strict liability” laws where *mens rea* is not required, but, interestingly, these laws are typically enacted in order to deter certain outcomes—such as the sale of alcohol to underage teenagers or the provision of unsafe working conditions in a factory—and are thus rooted in *control* rather than *truth* motives.
